# Challenges and opportunities for conducting a vaccine trial during the COVID-19 pandemic in the United Kingdom

**DOI:** 10.1177/17407745211024764

**Published:** 2021-06-22

**Authors:** M Estée Török, Benjamin R Underwood, Mark Toshner, Claire Waddington, Emad Sidhom, Katherine Sharrocks, Rachel Bousfield, Charlotte Summers, Caroline Saunders, Zoe McIntyre, Helen Morris, Jo Piper, Gloria Calderon, Sarah Dennis, Tracy Assari, Anita Marguerie de Rotrou, Ashley Shaw, John Bradley, John O’Brien, Robert C Rintoul, Ian Smith, Ed Bullmore, Krishna Chatterjee

**Affiliations:** 1 Department of Medicine, University of Cambridge, Cambridge, UK; 2 Departments of Infectious Diseases & Microbiology, Cambridge University Hospitals NHS Foundation Trust, Cambridge, UK; 3 Public Health England, Clinical Microbiology and Public Health Laboratory, Cambridge, UK; 4 Windsor Research Unit, Fulbourn Hospital, Cambridgeshire and Peterborough NHS Foundation Trust, Cambridge, UK; 5 Gnodde Goldman Sachs Translational Neuroscience Unit, Department of Clinical Neurosciences, University of Cambridge, Cambridge, UK; 6 Department of Respiratory Medicine, Royal Papworth Hospital, Cambridge, UK; 7 John V Farman Intensive Care Unit, Cambridge University Hospitals NHS Foundation Trust, Cambridge, UK; 8 NIHR Cambridge Clinical Research Facility, Cambridge Clinical Research Centre, Cambridge University Hospitals NHS Foundation Trust, Cambridge, UK; 9 Office for Translational Research, University of Cambridge, Cambridge, UK; 10 Research & Development Department, Cambridge University Hospitals NHS Foundation Trust, Cambridge, UK; 11 Medical Director’s Office, Cambridge University Hospitals NHS Foundation Trust, Cambridge, UK; 12 NIHR Cambridge Biomedical Research Centre, Cambridge, UK; 13 Department of Oncology, University of Cambridge, Cambridge, UK; 14 Institute of Metabolic Science, University of Cambridge, Cambridge, UK

**Keywords:** COVID-19, SARS-CoV-2, coronavirus, vaccine, clinical trial, pandemic

## Abstract

The COVID-19 pandemic has resulted in unprecedented challenges for healthcare systems worldwide. It has also stimulated research in a wide range of areas including rapid diagnostics, novel therapeutics, use of technology to track patients and vaccine development. Here, we describe our experience of rapidly setting up and delivering a novel COVID-19 vaccine trial, using clinical and research staff and facilities in three National Health Service Trusts in Cambridgeshire, United Kingdom. We encountered and overcame a number of challenges including differences in organisational structures, research facilities available, staff experience and skills, information technology and communications infrastructure, and research training and assessment procedures. We overcame these by setting up a project team that included key members from all three organisations that met at least daily by teleconference. This group together worked to identify the best practices and procedures and to harmonise and cascade these to the wider trial team. This enabled us to set up the trial within 25 days and to recruit and vaccinate the participants within a further 23 days. The lessons learned from our experiences could be used to inform the conduct of clinical trials during a future infectious disease pandemic or public health emergency.

## Background

Clinical trials are the cornerstone of modern medicine, providing scientific evidence to treat patients safely and effectively. In the context of novel drugs or vaccines, preclinical data generated in the laboratory and in animal models are used to inform and design clinical trials in humans. These include several phases of experimentation that progress sequentially from phase I studies, when the investigational medical product is tested for the first time in healthy human volunteers, through to phase III studies, in which the investigational medical product is tested for efficacy, effectiveness and safety in large numbers of subjects. Under normal circumstances, this process takes many years,^1^ and if successful, results in licencing of the drug or vaccine.

In the United Kingdom, the National Institute of Health Research, established in 2006 under the government’s health research strategy, sought to ‘create a health research system in which the National Health Service (NHS) supports outstanding individuals, working in world-class facilities, conducting leading-edge research focused on the needs of patients and the public’.^2^ The National Institute of Health Research is the nation’s largest funder of healthcare and social care research and works in partnership with the NHS, universities, local government, other research funders, patients and the public.

Since the emergence of SARS-CoV-2, the virus that causes COVID-19, in China in December 2019, it has infected over 115 million people and caused 2.5 million deaths.^3^ In the United Kingdom, there have been 4.2 million cases and 124,000 deaths as of 8 March 2021.^4^ The National Institute of Health Research has played a critical role in the fight against COVID-19, prioritising 85 urgent public health research studies and recruiting over 100,000 participants to clinical studies by June 2020.^5^ One of these trials is of a novel coronavirus vaccine, ChAdOx1 nCov-19, which was developed by the Oxford Vaccine Group in January 2020 and started phase I/II clinical trials in April 2020.^6^ Here, we describe our experience of rapidly setting up and conducting the phase III vaccine trial^7^ in Cambridgeshire, using existing staff and infrastructure across three NHS Trusts in the region. Our experiences were pertinent given the National Institute of Health Research’s intention to recruit over 100,000 participants to further COVID-19 vaccine trials in the United Kingdom.^8^

## Timetable of events

On 3 May 2020, one of the authors (M.E.T.) was invited to participate as a site in the COV002 trial by the University of Oxford. At this stage of the coronavirus pandemic, the three NHS Trusts in Cambridgeshire were focused on delivering clinical care to COVID-19 patients and the University of Cambridge had paused conduct of most non-essential, non-COVID-related research. Many research staff had been redeployed either to clinical roles or to conduct COVID-19-related research on inpatient wards.

To maximise the chance of demonstrating vaccine efficacy, the COV002 trial sought to recruit participants at greatest risk of exposure to SARS-CoV-2, including healthcare workers. To fulfil this aim, we decided to recruit healthcare workers from three NHS Trusts which encompassed different settings: emergency, inpatient and critical care (Cambridge University Hospitals NHS Foundation Trust); inpatient care and critical care (Royal Papworth Hospital); and mental health and community services (Cambridgeshire and Peterborough NHS Foundation Trust).

We quickly assembled a core study team that comprised one principal investigator from each of the three NHS Trusts, two Research and Development leads, four members of the National Institute of Health Research Cambridge Clinical Research Facility (Director, Director of Clinical Operations, Manager/Lead Research Nurse, Project Manager) and a project manager from the Office for Translational Research. Between 3 and 28 May 2020, we identified resources and facilities across the three NHS Trusts that could be deployed to conduct the trial, as shown in Table 1. Importantly, being aware of other ongoing COVID-19-related research and essential non-COVID research being undertaken on the sites, we identified research staff and resources that were not being fully utilised in other clinical trials.

**Table 1.** Organisations and resources.

Organisation / entity	Resources contributed
Cambridge University Hospitals NHS Foundation Trust (CUH)	Clinical medical and academic staff
	Clinical research nurses
	Administrative staff
	Phlebotomy staff
	Clinical trial pharmacy
	Occupational health
	Communications team
	Outpatient clinic for screening visits
Cambridgeshire and Peterborough NHS Foundation Trust (CPFT)	Clinical medical and academic staff
	Clinical research nurses and practitioners
	Windsor Research Unit
	Communications team
Royal Papworth Hospital (RPH)	Clinical medical and academic staff
	Clinical research nurses
	Communications team
NIHR Clinical Research Facility	Cambridge Clinical Research Centre Manager and Directors
	Research nurses
	Project management
	Administrative support
	Research space for vaccination clinics
	Laboratory facilities for sample processing and storage
	Storage space for a GMO IMP
Office for Translational Research	Project management
NIHR Clinical Research Network	Research nurses
Research Governance Committees	COVID-19 Research Oversight Group
	Cambridge Clinical Research Centre Scientific Advisory Board
	GMO Committee
	Clinical Trials Subcommittee
Research & Development Department	Joint agreements between Sponsor and NHS Trusts
	Research capacity and capability review
	Financial review
Training	Study specific – emails, slide sets, video calls
	Generic – local Trust/unit policies and procedures
Communications	Internal – email, Zoom, Slack, Google Docs
	External – email, MS Teams
	Publicity – Trust email, press releases, radio, TV
Security Department	Review of security arrangements
	Swipe card access for non-CUH staff
	Free parking for staff and participants

NHS: National Health Service; NIHR: National Institute for Health Research; GMO: genetically Modified Organism; IMP: investigational medicinal product.

Although unable to meet face-to-face, the study team communicated daily through video conferences to identify and assemble a large, diverse team of research staff from the three Trusts to set up and conduct the trial rapidly. This team comprised individuals working in different departments (e.g. infectious diseases, microbiology, respiratory medicine, intensive care medicine, psychiatry, metabolic medicine, clinical research nurses, research and development, pharmacy, occupational health, communications, security), many of whom were not acquainted with each other and who had never worked together. A full list of the study team and their roles is available at https://cambridge.crf.nihr.ac.uk/our-research/covid-19-oxford-vaccine-trial/. We also identified an outpatient clinic (usually used for plastic surgery clinics) to conduct screening visits and a clinical research area in the National Institute of Health Research Clinical Research Facility (usually used for paediatric clinical research studies) to conduct vaccination and follow-up visits.

On 8 May 2020, the trial was submitted to the local COVID-19 Research Oversight Group which had responsibility for oversight of all COVID-19-related research. The trial was also considered by other local regulatory bodies including the Scientific Advisory Board of the Cambridge Clinical Research Centre, the Genetically Modified Organism Committee and a subcommittee of the Clinical Trials Unit and by the Research and Development Departments of each NHS Trust. The principal investigator (M.E.T.) at Cambridge University Hospitals NHS Foundation Trust was responsible for preparing and submitting the study documents to these Committees. In parallel, the study protocol underwent a number of protocol amendments, each of which required review and approval by national regulatory bodies such as National Research Ethics Service, the Health Research Authority and Medical and Healthcare Regulatory Agency. These submissions were co-ordinated by the study sponsor (University of Oxford) and then reviewed and approved by local research and development staff in Cambridge prior to implementation by the study team. During this time period, we assembled a dedicated and enthusiastic research team of over 70 staff who planned the logistics of delivering the trial collaboratively between the three Trusts. The study team continued to meet once or twice a day by video conference during the study set-up period. Close liaison with the sponsor (University of Oxford) was critical to success in providing our team with expertise in diverse, requisite skill sets such as data entry into an on-line electronic database, storage and handling of genetically modified viral vaccine, and vaccine administration procedures. This involved weekly briefings by video conference between the sponsor and principal investigators at the UK study sites, which was cascaded to the study team. Additional training was provided by video conference with accompanied provision of study documents and training slides. Each member of the study staff completed and maintained a study training log to confirm that they had completed the requisite training. These were stored in the site study file and available for inspection by the sponsor and the Medical and Healthcare Regulatory Agency, as required.

On 28 May 2020, having secured all national and local regulatory approvals, the COV002 trial opened for recruitment of participants in Cambridge. The core study team advertised the trial to potential participants through numerous channels including email invitations and bulletins from the three NHS Trusts, the East of England Ambulance Service NHS Trust and the NHS Cambridgeshire and Peterborough Clinical Commissioning Group. We worked with communications teams at the three NHS Trusts and the University of Cambridge to issue press releases. We also publicised the trial through professional and personal social media channels (e.g. Twitter and Facebook) and participated in local radio and television interviews (BBC Radio Cambridgeshire and BBC Look East).

The clinicians and nurses in the study team started telephone screening on 29 May 2020, face-to-face screening clinics on 1 June 2020 and vaccination clinics on 10 June 2020. Over 1000 volunteers expressed an interest in taking part in the trial by completing an on-line questionnaire on the Cambridgeshire site of the Oxford vaccine trial portal. The questionnaire responses were downloaded from the website twice a day by a project manager and triaged by one of the principal investigators (M.E.T.). After triaging potential participants who fulfilled the initial screening criteria (healthcare workers aged 18–55 years), the study doctors and research nurses then conducted telephone screening to assess the eligibility of 554 individuals. Face-to-face screening, including serological testing of 427 individuals, was conducted between 1 and 8 June 2020. A total of 350 participants were eligible for vaccination, of which 304 participants were vaccinated between 10 and 20 June 2020. All screening and vaccination clinics were conducted with social distancing measures in green areas, and staff and participants wore appropriate personal protective equipment, supplied by the NHS Trusts. The timeline of events is summarised in Supplementary Figure 1.

## Challenges encountered and potential solutions

Having set up and conducted this trial rapidly, we have gained many insights which will enable us to be better prepared to deliver clinical research or trial intervention in the event of a second wave of COVID-19 infections or similar public health emergency. Unless completely unavoidable, we suggest that it is undesirable to redeploy all research facility capacity or staff to purely clinical roles in a pandemic or similar health emergency. Redeployment led some clinical departments to become over-staffed during the early days of the pandemic. Given the importance of research in combating the unprecedented COVID-19 pandemic, availability of dedicated research facility capacity and skilled research staff who could be deployed quickly and flexibly to deliver COVID-19 research, including this vaccine trial, proved invaluable.

From a practical standpoint, we identified a number of challenges which arose from the complexity of delivering a genetically modified organism vaccine trial across different organisations. Each organisation had its own research facilities and staff, different information technology and communication systems, and different standard operating procedures and methods for training and competency assessment. We rapidly assembled a strong project management team (that included representatives from all three organisations) which met daily by video conference to plan strategy and review progress. We identified a nursing lead at each organisation who worked together with the project management leads to identify available clinic space and staffing requirements and to collate training documents and competency assessments for trial staff. We were fortunate to have an on-site clinical research facility (experienced in running early-phase genetically modified organism trials) which had available capacity to host the vaccine trial, as other non-COVID studies had been temporarily suspended. We also identified clinic spaces that could be used for socially distanced screening and follow-up clinics. One of the most challenging aspects was that each organisation had different information technology systems, approved software packages and information governance procedures. This made communications and data sharing more difficult than it might otherwise have been, as we had to apply for information governance approval at each site to enable the trial staff to access the necessary software packages (Dropbox, Slack, Microsoft Teams) and the on-line clinical trial database (RedCAP). We initially set up a University Dropbox folder in order to collate and share study documents and then transitioned this to a Master study file on an NHS Trust server, with site folders at the other two NHS sites. We used Zoom video conferences for daily team meetings and a combination of email and Slack for written communications. We used the secure nhs.net email service to transfer patient-identifiable information between different NHS sites. External training was provided by the sponsor using Microsoft Teams, as previously described, and was cascaded internally using videos, slide sets or small-group socially distanced, face-to-face training sessions. From a research governance perspective, we had to seek approval from several different committees sequentially prior to obtaining final approval by the COVID-19 Research Oversight Group; this was time-consuming and could have been made easier by a parallel submission process. Despite these challenges, we managed to open the trial to recruitment only 25 days after being invited to participate as a site. This was remarkable given that this process can take several months under normal circumstances. Table 2 summarises the challenges that we encountered and suggests potential solutions to these.

**Table 2.** Challenges encountered and potential solutions.

Item	Challenge	Solution(s)
Research governance	Complex committee structures with need for multiple separate reviews	Single committee with wide representation from all agencies required to review and approve a study protocol at one sitting
Research facilities	Research facilities on several different sites	Central inventory of research facilities, (including capabilities and availability) so that they can be identified and used effectively, when required
Research staff	Multiple research teams on several different sites	Central inventory of research staff (including training, capabilities and availability) so that they can be deployed most effectively when required Rapid process to issue research passports to enable staff to work across different sites and NHS Trusts Flexibility in HR and funding arrangements to enable staff to be deployed rapidly into clinical trial teams
Vaccine storage and handling	GMO vaccine	Standardised policy for storage and handling GMO products that can be modified for each trial A GMO Committee that can meet ad hoc to rapidly approve GMO trial protocols/policies Appropriate storage facilities for GMO products
SOP	Different standard operating procedures for similar clinical research procedures between sites	Standardised SOPs for common clinical research procedures which could be used by all sites collaborating to undertake a research project
Training procedures	Multiple different training documents for similar training requirements between sites	Standardised training procedures for common skills which could be used by all sites involved in collaborative research projects
Competency assessment	Multiple different methods/documents forcompetency assessment of training between sites	Standardised competency assessments and documentation which could be used by all sites involved in collaborative research projects
IT infrastructure and governance	Multiple different IT systems and softwareapplicationsbetween different sites in Cambridge and atother UK centres	A study folder on an NHS server, accessible by all relevant members of the study team from multiple sites Information governance approval for all study members to be able to use software applications (e.g. Microsoft Teams, REDCap) required to operate electronic CRFs
Communication platforms	Multiple different communication platforms atdifferent sites and lack of compatibilitybetween them Difficulty in transferring large files by email	Use of daily video calls to communicate with the teams at different sites Use of Slack for within-team communication to reduce email traffic and to share documents Use of NHS.net emails to transfer person-identifiable information between different NHS sites Use of a file sharing system, for example, Dropbox, to share large files between sites

NHS: National Health Service; GMO: genetically modified organism; SOP: standard operating procedure; CRF: case report form.

## Insights gained and suggestions for the future

Our experience provides a number of useful insights for future conduct of translational research in the United Kingdom. Nevertheless, we think that some of our experiences and the lessons learned could be adapted to other research settings. We do recognise, however, that there may be additional challenges in different settings such as in low- and middle-income countries (where clinical trials infrastructure may be less well developed) and in high-income countries with different regulatory requirements. First, in a public health emergency, it is possible to safely complete the research governance process (i.e. obtaining the necessary national and local approvals to conduct the trial) in an expedited manner. Second, none of the resources used to deliver this trial were established with the purpose of delivering clinical research in the context of an infectious disease pandemic, yet all proved capable of doing so. This vividly illustrates the generic and transferable nature of clinical research skills, enabling their flexible and rapid redeployment in a time of health crisis. It would not have been possible to deliver this trial without previous National Institute of Health Research and NHS investment in both physical and human research infrastructure. For example, the National Institute of Health Research Cambridge Clinical Research Facility at Cambridge University Hospitals NHS Foundation Trust was established to deliver complex, small-scale studies in experimental medicine. Similarly, the Windsor Research Unit (Cambridgeshire and Peterborough NHS Foundation Trust), a key clinical site for this study, is supported to deliver clinical research in dementia and related areas of mental and physical health. Research infrastructure and staff at Royal Papworth Hospital support studies in cardiopulmonary medicine. With strong leadership and appropriate training, research staff did not hesitate to undertake unfamiliar study protocols in high-risk clinical environments. Similarly, striking cooperation across institutional boundaries has allowed rapid and successful collaboration to deliver vital research in the public interest. We have benefitted from the support of senior leadership within our organisations who appreciated the potential significance of this trial. Our experience highlights the importance of investment in sustainable clinical research infrastructure that is ready to respond at a moment’s notice to face this or future emergent infectious public health emergencies or pandemics.

## Supplemental Material

sj-pdf-1-ctj-10.1177_17407745211024764 – Supplemental material for Challenges and opportunities for conducting a vaccine trial during the COVID-19 pandemic in the United Kingdom Supplemental material, sj-pdf-1-ctj-10.1177_17407745211024764 for Challenges and opportunities for conducting a vaccine trial during the COVID-19 pandemic in the United Kingdom by M Estée Török, Benjamin R Underwood, Mark Toshner, Claire Waddington, Emad Sidhom, Katherine Sharrocks, Rachel Bousfield, Charlotte Summers, Caroline Saunders, Zoe McIntyre, Helen Morris, Jo Piper, Gloria Calderon, Sarah Dennis, Tracy Assari, Anita Marguerie de Rotrou, Ashley Shaw, John Bradley, John O’Brien, Robert C Rintoul, Ian Smith, Ed Bullmore and Krishna Chatterjee in Clinical Trials

## Appendix 1

**Cambridge COVID Vaccine Trial Team**

M. Estée Török; Benjamin R Underwood; Mark Toshner; Claire Waddington; Emad Sidhom; Katherine Sharrocks; Rachel Bousfield; Charlotte Summers; Krishna Chatterjee; Caroline Saunders; Zoe McIntyre; Helen Morris; Jo Piper; Carla Ribeiro; Lorinda Pickup; Ranalie de Jesus; Karen Brookes; Phoebe Vargas; Caroline McMahon; Chiara Macor; Ciro Pasquale; Areti Bermperi; Evgenia Kourampa; Marivic Fabiculcana; Julie Ann Zerudo; Vivien Mendoza; Stewart Fuller; Claire Glemas; Sharon Baker; Greta Lyons; Gloria Calderon; Marina Bishop; Siobhan Coleman; Naomi Thomas; Vince Mlilo; Edward Stanton; Rachel Michel; Julie Philips; Heidi Rice; Sarah Dennis; Lucie Garner; Francesca Rosa; Saji Victor; Julie Zamikula; Sue Mepham; Fatima Hajee; Juan Carlos Quijano-Campos; Laura Watson; Christopher Georgiou; Jieniean Worsley; Djamilla Shamtally-Amode; Ferishta Rahimi; Heather Jones; Isabel Cruz; Gayle Lindsay; Christian Sparke; Anna Chapman; Katie Keating-Fedders; Codie Fahey; Danielle Johnson; Lucy Worboys; Simone Hargreaves; Ashlea Bucke; Lindsay Carr; Jackie Hampshire; Tracy Richardson; Jenny Sharpe; Lynne Whitehead; Naval Vyse; Stephen Kelleher; Tracy Assari; Mary-Beth Sherwood; Vicki Hughes; Kim Giraud.
