# Supplementary figures and images for: Challenges and opportunities for conducting a vaccine trial during the COVID-19 pandemic in the United Kingdom
Source: Clin Trials. 2021 Jun 22;18(5):615–21. doi: 10.1177/17407745211024764 (PMC8479147; doi:10.1177/17407745211024764)

## Supplementary Figure 1. Timeline of events

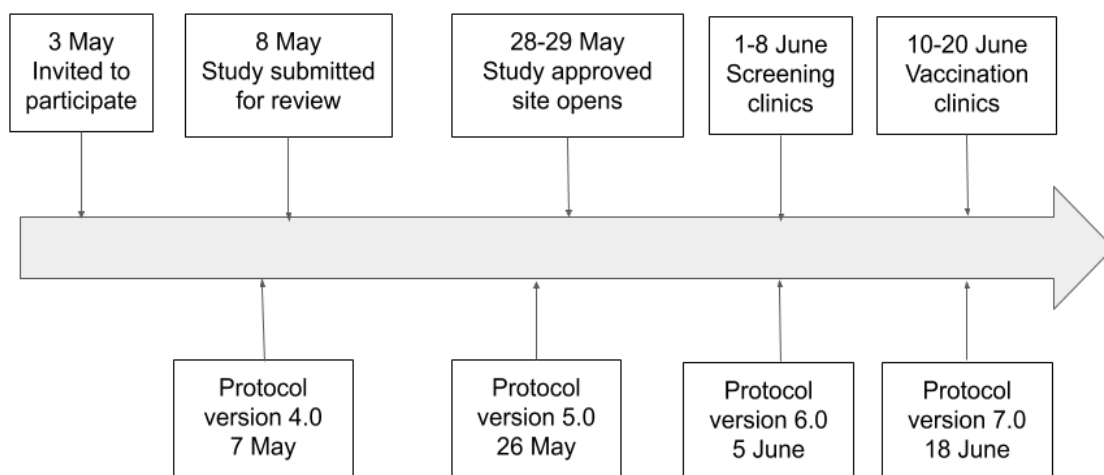

Supplement: sj-pdf-1-ctj-10.1177_17407745211024764 – Supplemental material for Challenges and opportunities for conducting a vaccine trial during the COVID-19 pandemic in the United Kingdom [file sj-pdf-1-ctj-10.1177_17407745211024764.pdf]
